# The Effects of a Low GI Diet on Cardiometabolic and Inflammatory Parameters in Patients with Type 2 and Gestational Diabetes: A Systematic Review and Meta-Analysis of Randomised Controlled Trials

**DOI:** 10.3390/nu11071584

**Published:** 2019-07-12

**Authors:** Omorogieva Ojo, Osarhumwese Osaretin Ojo, Xiao-Hua Wang, Amanda Rodrigues Amorim Adegboye

**Affiliations:** 1 Faculty of Education and Health, Department of Adult Nursing and Paramedic Science, University of Greenwich, London SE9 2UG, UK; 2 South London and Maudsley NHS Foundation Trust, University Hospital, Lewisham High Street, London SE13 6LH, UK; 3 The School of Nursing, Soochow University, Suzhou 215006, China; 4 Faculty of Education and Health, Department of Psychology, Social Work & Counselling, University of Greenwich, London SE9 2UG, UK

**Keywords:** type 2 diabetes, gestational diabetes, glycemic index, randomised controlled trial, lipid profile, inflammatory parameters

## Abstract

The prevalence of diabetes is increasing globally, and its effect on patients and the healthcare system can be significant. Gestational diabetes mellitus (GDM) and type 2 diabetes are well established risk factors for cardiovascular disease, and strategies for managing these conditions include dietary interventions, such as the use of a low glycemic index (GI) diet. Aims: This review aimed to evaluate the effects of a low GI diet on the cardio-metabolic and inflammatory parameters in patients with type 2 diabetes and women with GDM and assess whether the effects are different in these conditions. Methods: This review was based on the preferred reporting items for systematic reviews and meta-analyses (PRISMA) guidelines. Three databases (EMBASE, Pubmed, and PsycINFO) were searched from inception to 20 February 2019 using search terms that included synonyms and Medical Subject Headings (MeSH) in line with the population, intervention, comparator, outcomes, and studies (PICOS) framework. Studies were evaluated for the quality and risk of bias. Results: 10 randomised controlled studies were included in the systematic review, while 9 were selected for the meta-analysis. Two distinct areas were identified: the effect of a low GI diet on lipid profile and the effect of a low GI diet on inflammatory parameters. The results of the meta-analysis showed that there were no significant differences (*p* > 0.05) between the low GI and higher GI diets with respect to total cholesterol, HDL, and LDL cholesterol in patients with type 2 diabetes. However, there was a significant difference (*p* = 0.027) with respect to triglyceride which increased by a mean of 0.06 mmol/L (0.01, 0.11) in patients with type 2 diabetes on higher GI diet. With respect to the women with GDM, the findings from the systematic review were not consistent in terms of the effect of a low GI diet on the lipid profile. The results of the meta-analysis did not show significant differences (*p* > 0.05) between low GI and higher GI diets with respect to adiponectin and C-reactive proteins in patients with type 2 diabetes, but a significant difference (*p* < 0.001) was observed between the two groups in relation to interleukin–6. Conclusion: This systematic review and meta-analysis have demonstrated that there were no significant differences (*p* > 0.05) between the low GI and higher GI diets in relation to total cholesterol—HDL and LDL cholesterol—in patients with type 2 diabetes. However, a significant difference (*p* < 0.05) was observed between the two groups with respect to triglyceride in patients with type 2 diabetes. The results of the effect of a low GI diet on the lipid profile in patients with GDM were not consistent. With respect to the inflammatory parameters, the low GI diet significantly decreased interleukin–6 in patients with type 2 diabetes compared to the higher GI diet. More studies are needed in this area of research.

## 1. Introduction

Globally, there is an increasing prevalence of diabetes, with over 420 million people living with the condition. This number has significant implications for health care provisions due to the impact of diabetes and its complications on those who have the condition [1,2]. Type 2 diabetes is usually characterised by insulin deficiency due to beta cell dysfunction and often involves insulin resistance [3]. On the other hand, hyperglycaemia first detected at any time during pregnancy is classified either as diabetes in pregnancy or Gestational Diabetes Mellitus (GDM), and are usually diagnosed based on the fasting and/or 2 h plasma glucose following a 75 g oral glucose load [4].

Both type 2 diabetes and GDM have implications for carbohydrate, protein and fat metabolism and may predispose individuals to acute and long-term complications [5]. About half of the women diagnosed with GDM proceed to develop type 2 diabetes within 5 to 10 years after giving birth [6]. Due to changing lifestyles, type 2 diabetes is increasingly diagnosed in children [7]. In 2013, over 3.2 million adults were diagnosed with diabetes in England and Wales, with prevalence rates of 6% and 6.7% in England and Wales, respectively [7]. In addition, about 90% of adults who are currently diagnosed with diabetes have type 2 diabetes, with the burden of the disease disproportionally affecting ethnic minorities, particularly Africans, African-Caribbeans, and South Asians [7].

GDM presents adverse risks to the mother and child during the pre- and post–natal period [4]. It was estimated that 21.3 million live births had some form of raised blood glucose or hyperglycaemia in 2017, and about 85.1% of these were due to GDM [6]. This represented about one in every seven births affected by GDM [6]. In England and Wales, of the estimated 700,000 women who give birth every year, about 5% have either pre-existing diabetes or GDM [8]. About 87.5% of these women who have diabetes during pregnancy have GDM [8]. Therefore, management strategies, including dietary interventions such as the use of a low glycemic index (GI) diet, have been recommended instead of a higher GI diet, in order to manage hyperglycaemia and mitigate related complications [8]. In our previous review on the effect of a low GI diet in patients with type 2 diabetes, Ojo et al. [9] found that a low GI diet was more effective in controlling glycated haemoglobin and fasting blood glucose compared with a higher GI diet in these patients. Therefore, this current review builds on the earlier systematic review and meta-analysis [9] by assessing the impact of a low GI diet on lipid profile and inflammatory markers.

Why is it important to do this review? GDM is closely associated with type 2 diabetes, as they share many key pathophysiological characteristics including progressive insulin resistance [3,10]. In addition, people who develop either type 2 diabetes or GDM have similar risk factors, such as ethnicity (South Asian or Afro–Caribbean), a high body mass index (BMI), family history, and advanced age [5,10].

Although low grade inflammation and insulin resistance are part of normal physiological adaptation of pregnancy, these processes are exacerbated in patients with GDM and obesity [11]. Elevated levels of inflammatory components, such as tumour necrosis factor alpha (TNF-α), have been shown to correlate with progressive insulin resistance in pregnancy and are associated with hyperinsulinaemia in obesity and in patients with type 2 diabetes [10]. The risks associated with GDM, including postpartum type 2 diabetes, increase with progressive hyperglycaemia [8,12]. Furthermore, both GDM and type 2 diabetes are well established risk factors for cardiovascular diseases [3,5,13].

This calls for scrutiny and a greater understanding of the role of low GI diets on inflammatory parameters and lipid profiles (cardiometabolic parameters) in these patients, as the biomarkers have implications for insulin resistance and cardiovascular mortality. We know this based on the knowledge that dietary interventions are useful approaches to managing type 2 diabetes and GDM. Therefore, a study should involve an evaluation of the effect, and quality and quantity of the macro and micronutrients in the foods consumed. Of particular interest is the quality of carbohydrate in the diets of people with type 2 diabetes or GDM, often linked to its glycemic index (GI). Foods with low GI may improve glycemic control including the reduction in glycated haemoglobin (HbA1c) through improvement in peripheral insulin sensitivity [14,15,16,17,18,19]. However, the evidence regarding the effect of a low GI diet on lipid profiles is still conflicting.

Bouchie et al. [20] revealed that 5 weeks of a low GI diet was useful in improving plasma lipids in non-diabetic men who were moderately overweight. However, in normolipidemic well controlled patients with type 2 diabetes, Brand et al. [21] observed that low GI diets did not provide improvement in plasma lipids. Clar et al. [22], in their meta–analysis, noted that there is, presently, no compelling evidence that shows that low GI diets have significant beneficial effects on blood lipids. On the other hand, Schwingshacki and Hoffman [23] demonstrated that a low GI diet has beneficial effects with respect to pro–inflammatory markers, such as C–reactive protein (CRP) which may be useful in preventing obesity associated diseases. This study involved both patients who had type 2 diabetes and participants who were non–diabetic. The systematic reviews by Goff et al. [13], Clar et al. [22], and Fleming and Godwin [24] were based on assessing the effect of low GI diets on lipid profiles on either general participants or those with cardiovascular diseases. No previous review has assessed the effect of a low GI diet on both lipids and the inflammatory profile of patients with type 2 diabetes and/or GDM. Therefore, the current review evaluates the impact of a low GI diet on the cardio-metabolic and inflammatory parameters in patients with type 2 diabetes and GDM. This is based on the understanding that the control of cardio-metabolic parameters is a useful approach in managing patients with type 2 diabetes and women with GDM [4,25].

Objectives:

This is a systematic review and meta-analysis which:

- Evaluates the effect of a low GI diet on cardio-metabolic and inflammatory parameters in patients with type 2 diabetes and women with GDM.

## 2. Methods

This systematic review and meta-analysis was written according to the preferred reporting items for systematic reviews and meta-analyses (PRISMA) guidelines [26]. The eligibility criteria for paper inclusion according to type of study, participants, intervention, and outcomes are described below.

Types of Studies:

Randomised controlled studies were the only studies included in this review (Table 1 and Table 2).

Type of Participants:

Patients with type 2 diabetes or pregnant women with gestational diabetes were the participants of interest in all the studies selected (Table 2).

Type of Interventions:

Diets with low GI were compared with diets with higher GI in patients with type 2 diabetes and in women with GDM. The classification of diets as having either low GI or higher GI was based on the lower GI values of the intervention diets (low GI diet).

### 2.1. Outcomes of Interest

The primary measures of interest were:

- Cardio-metabolic parameters: total cholesterol (TC) mmol/L, low density lipoprotein (LDL) cholesterol mmol/L, high density lipoprotein (HDL) cholesterol mmol/L, and triglycerides (TG) mmol/L.

The secondary outcome measures were:

- Inflammatory parameters: C–reactive protein (CRP) mg/L, Adiponectin mg/L, and Interleukin–6 (IL-6) mg/L.

### 2.2. Search Terms and Search Strategy

The process of searching for articles for this review relied on the Population, Intervention, Comparator, Outcome, and Study design (PICOS) approach [27] and involved electronic databases (EMBASE, Pubmed, and PsycINFO) from inception to 20 February 2019. A number of articles were identified through this process by using search terms, including Medical Subject Headings (MeSH) and synonyms. Boolean operators (AND/OR) were used to combine words and search terms (Table 1). The reference lists of included articles were manually searched for relevant papers.

### 2.3. Inclusion and Exclusion Criteria

**Figure 1:**
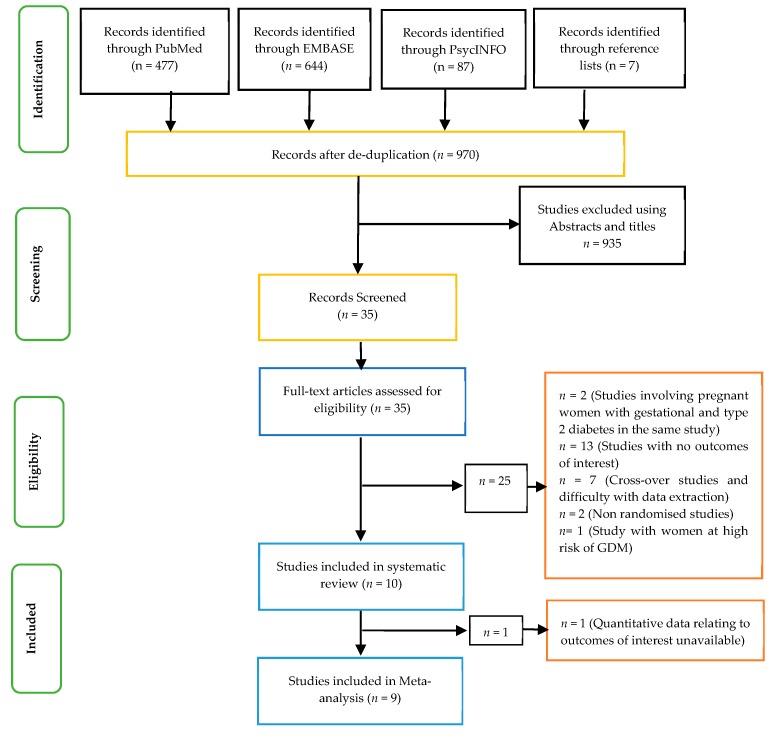
Prisma flow chart showing the studies included.

The criteria for selecting studies are outlined in Table 2. No time or language restriction was applied. Only primary research studies that were randomised controlled trials were selected for this review. In addition, studies involving patients with type 2 diabetes or GDM and the use of low GI diets across the world were included. Those studies not meeting the criteria set out in Table 2 and the text were excluded from this review. In this regard, studies that had animals, patients with type 1 diabetes, children with diabetes, or healthy adults without diabetes were excluded from the current review (Table 2). In addition, observational studies and those involving dietary supplements were excluded. Therefore, a total of 9 studies were included in the meta-analysis (Figure 1).

### 2.4. Quality Assessment and Risk of Bias of Included Studies

The Critical Appraisal Skills Programme (CASP) checklist for randomised controlled trials [28] was used to evaluate the studies. In addition, the Cochrane risk of bias tool [29,30] was used to assess the methodological quality of the included studies. A grade (or score) was allocated to each trial on the basis of selection bias, performance bias, detection bias, attrition bias, and reporting bias. This process involved reviewing details about the similarity at baseline of the groups being compared, the blindness of the outcome measurement and participants, the randomisation method, dropout rates, selective reporting, and compliance with the intervention. On the basis of this information, studies were categorised into three groups: (a) low risk of bias, (b) unclear risk of bias, and (c) high risk of bias.

### 2.5. Data Extraction and Management

#### Statistical Analysis

Treatment effects were summarized as the weighted mean difference (WMD) with standard deviation by using the absolute change values from baseline to post-intervention for control and intervention groups. The meta-analysis was performed in stata (version 15.0, Stata Corp, College Station, TX, USA). Fixed-effects models were applied to estimate the overall weighted mean difference.

All results were presented with a 95% confidence interval (CI) and displayed on a forest plot and table, and the null hypothesis of no effect was rejected at *p* ≤ 0.05. In addition to the forest plots, I^2^ statistics were assessed to quantify the degree of heterogeneity. Values <25% were considered to be low, 25%–50% moderate, and >50% high. *Q* statistics was also used to assess heterogeneity. The null hypothesis of homogeneity was rejected if *p* < 0.1, given the low power of the test [29].

### 2.6. Data Inclusion Decisions

Gomes et al. [31] expressed their results as the median and interquartile ranges, which were converted to means and standard deviations [29]. In addition, for the meta-analysis, the units of measurements for the lipid parameters were converted to mmol/L, while for the inflammatory markers, they were converted to mg/L. The Grant et al. [32] study was not included in the meta-analysis, as the information provided showed that there were no significant differences (*p* > 0.05) between the low GI and the higher GI groups in terms of lipids and inflammatory markers. However, these results were not expressed in quantitative terms.

## 3. Results

Ten studies were selected for the systematic review (Table 3), and nine studies were included in the meta-analysis (Table 4). In addition, while four of the studies were conducted in Canada, two were carried out in China and one study each was carried out in Brazil, Greece, Malaysia, and the USA.The total number of subjects in the eight studies included in the meta-analysis in patients with type 2 diabetes involved 394 participants in the low GI group and 388 participants in the higher GI group. There were 41 subjects in the low GI group compared with 42 participants in the higher GI group in the only study on women with GDM. 

Based on the systematic review (Table 3 and Table 4) and meta-analysis, two distinct areas have been identified: the effect of a low GI diet on lipid profiles and the effect of a low GI diet on inflammatory parameters.

### 3.1. Evaluation of the Risk of Bias of the Studies Selected

**Figure 2:**
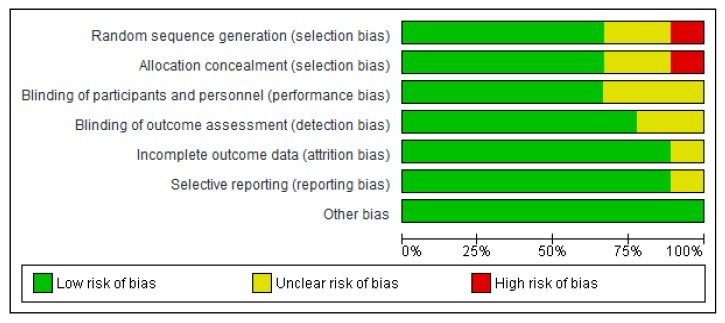
A summary risk of bias graph of included studies.

**Figure 3:**
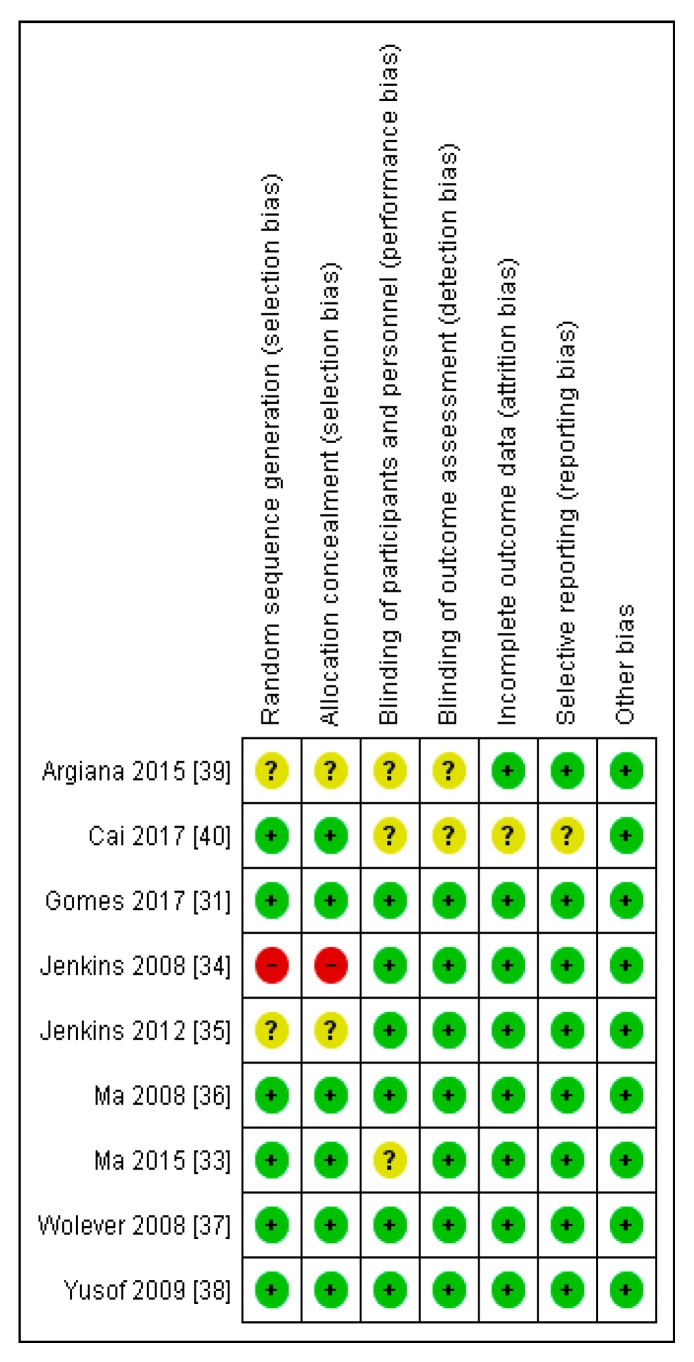
A risk of bias graph for each included study.

Most of the studies demonstrated either a low risk of bias or an unclear risk of bias in all the domains evaluated (selection bias, performance bias, detection bias, attrition bias, and reporting bias) (Figure 2). However, Jenkins et al. [34] showed high risk of bias in the area of selection bias (Figure 3).

### 3.2. The Effect of a Low GI Diet on Lipid Profile

Grant et al. [32] found no significant differences (*p* > 0.05) between the low GI group compared to the higher GI group, with respect to the lipid profile in women with GDM (Table 3). In their study, Ma et al. [33] observed that the increases in total cholesterol (0.12 versus 0.23 mmol/L) and triglyceride (0.41 versus 0.56 mmol/L), and the decrease in HDL cholesterol (−0.01 versus −0.11), were significantly lower (*p* < 0.05) than the higher GI group in women with GDM (Table 4).

**Figure 4:**
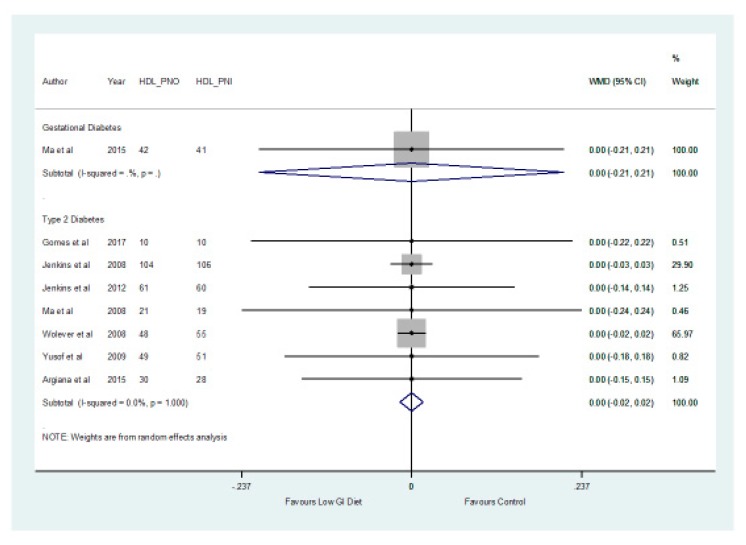
A forest plot showing the effects of a low GI diet on HDL cholesterol (mmol/L).

**Figure 5:**
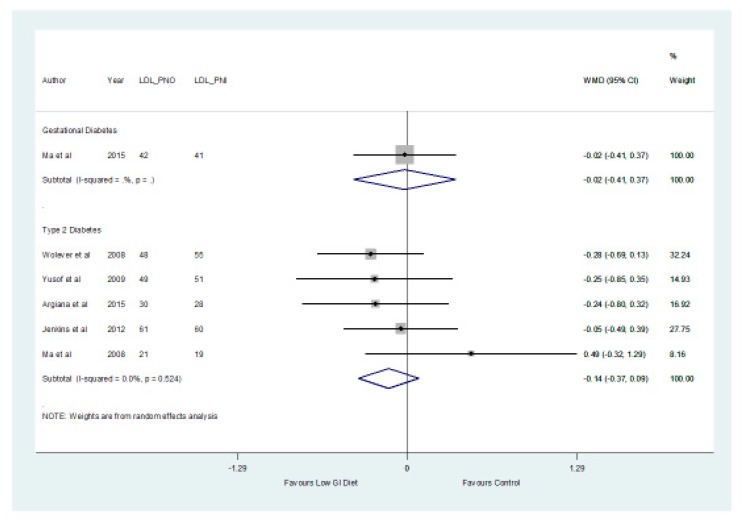
A forest plot showing the effect of a low GI diet on LDL cholesterol (mmol/L).

In patients with type 2 diabetes, the results of the meta-analysis showed no significant differences (*p* > 0.05) between the low GI and higher GI groups with respect to HDL with a mean difference of 0.00 mmol/L (−0.02, 0.02) and LDL cholesterol with a mean difference of −0.14 mmol/L (−0.37, 0.09) (Figure 4 and Figure 5, respectively).

**Figure 6:**
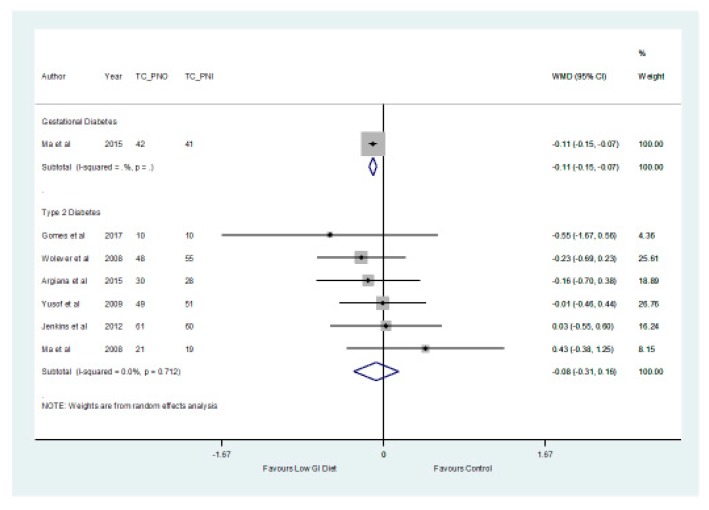
A forest plot depicting the effect of a low GI diet on Total Cholesterol (mmol/L).

**Figure 7:**
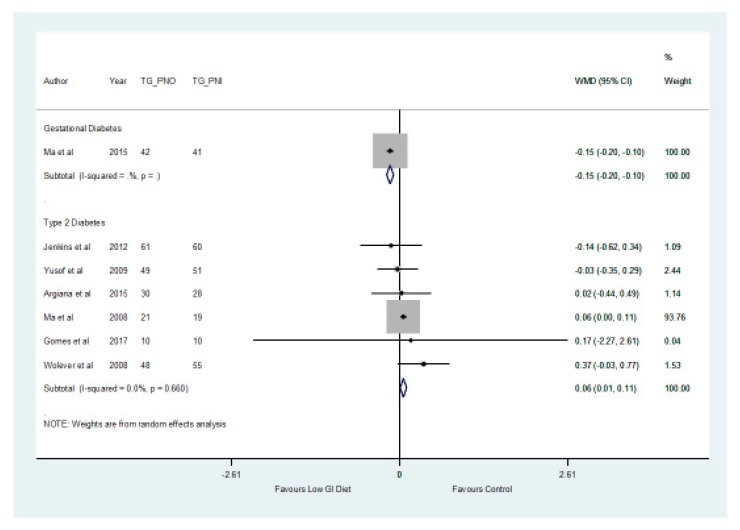
A forest plot depicting the effect of a low GI diet on Triglyceride (mmol/L).

In addition, the findings from the meta-analysis found no significant difference (*p* > 0.05) between the two groups in relation to the total cholesterol which decreased by a mean of −0.08 mmol/L (−0.31, 0.16) (Figure 6) in the low GI group. The results showed that there was a significant difference (*p* = 0.027) with respect to triglycerides, which increased by a mean of 0.06 mmol/L (0.01, 0.11) in patients with type 2 diabetes in the higher GI group (Figure 7).

### 3.3. The Effect of a Low GI Diet on Inflammatory Parameters

According to Grant et al. [32], there was no significant difference (*p* > 0.05) between the low GI and higher GI groups in relation to the C–reactive protein in women with GDM. In patients with type 2 diabetes, Gomes et al. [31] and Cai et al. [40] found that a low GI diet can reduce or prevent the inflammatory responses induced by a high GI diet. In addition, a low GI diet has been shown to reduce C–reactive protein levels significantly compared to a high GI diet [37,39].

With respect to serum interleukin–6 and adiponectin, Argiana et al. [39] did not find significant differences in both groups between week 0 and week 12 in patients with type 2 diabetes. The results of the meta-analysis did not show significant differences (*p* > 0.05) between low GI and higher GI diets with respect to adiponectin and C-reactive protein in patients with type 2 diabetes (Table 5). However, a significant difference (*p* < 0.001) was observed between the two groups in relation to interleukin–6 (Table 5), with the low GI diet decreasing interleukin–6 by a mean of −1.01 mg/L (−1.55, −0.48). A meta-analysis was not conducted for the patients with GDM with respect to inflammatory parameters due to the limited number of studies.

## 4. Discussion

The results of the two studies [32,33] that evaluated the effects of low GI diets on lipid profiles in women with GDM were not consistent. While Grant et al. [32] did not find significant differences (*p* > 0.05) between the low GI and higher GI groups in relation to lipids, Ma et al. [33] found significant differences (*p* < 0.05) between the two groups with respect to total cholesterol, triglycerides, and HDL cholesterol. On the other hand, the results of the meta-analysis showed that there were no significant differences (*p* > 0.05) between the low GI diet and higher GI group with respect to total cholesterol, HDL, and LDL cholesterol in patients with type 2 diabetes (although the difference was statistically significant (*p* < 0.05) in relation to triglycerides, with higher GI diet increasing triglyceride levels). The differences observed between the effects of a low GI in patients with GDM compared to patients with type 2 diabetes in some of the metabolites may due to the limited number of studies in the current review and the differences in the pathophysiology of both conditions. In a previous meta-analysis, Fleming and Godwin [24] revealed that a low GI diet may help lower total cholesterol and LDL cholesterol. In addition, Goff et al. [13] found that low GI diets reduced total and LDL cholesterol and had no effect on HDL cholesterol and triglycerides. It is possible that the differences between the current review and the previous reviews in relation to some of the metabolites may be due to the participants included in the studies. While this review was based only on patients with GDM and type 2 diabetes, the earlier reviews were based on the general population [24] or included participants without diabetes [13].

The results of the effect of a low GI diet on inflammatory markers were variable in the studies selected in patients with GDM. However, the results of the meta-analysis showed that differences between low GI and higher GI groups were only significant (*p* < 0.05) in relation to interleukin–6, which decreased in the low GI group in patients with type 2 diabetes. These results are discussed below.

### 4.1. The Effect of a Low GI Diet on Lipid Profile

There appears to be controversy regarding the role of low GI diets in the prevention of cardiovascular diseases. The effect of GI on total cholesterol, LDL, and HDL cholesterol is not quite clear [39,41]. For example, in women with GDM, Grant et al. [32] reported no significant differences (*p* > 0.05) between the low GI and higher GI groups with respect to the lipids in women with GDM. However, Ma et al. [33] found that low GI diets improved blood lipids.

In patients with type 2 diabetes, Gomes et al. [31] found that a low GI diet reduced body fat. In the study by Jenkins et al. [34], it was observed that HDL cholesterol increased by 1.7 mg/dL in the low GI group and decreased by −0.2 mg/dL in the higher GI group (*p* = 0.005). In contrast, Wolever et al. [37] noted that HDL cholesterol was 4% lower in the low GI group, while the higher GI values were intermediate. Other studies [35,36,38] demonstrated no significant difference between the low GI and higher GI groups in relation to HDL cholesterol.

According to Jenkins et al. [35], low GI legumes produced significant decreases in total cholesterol level (*p* < 0.001) in patients with type 2 diabetes. The relative reduction in total cholesterol level was greater in the low GI legume diet group compared with the higher GI group [35]. However, Ma et al. [36] and Wolever et al. [37] did not find a significant difference between low GI and higher GI diets with respect to the total cholesterol in patients with type 2 diabetes.

Jenkins et al. [35] demonstrated that a low GI legume produced significant decreases in triglycerides (*p* < 0.001) in patients with type 2 diabetes. Decreases in triglycerides in the low GI group were also reported by Yusof et al. [38], although Wolever et al. [37] showed increased levels of triglycerides in the low GI group. On the other hand, Ma et al. [36] found no significant difference between low GI and higher GI with respect to triglycerides.

The mechanism by which dietary GI influences blood lipids has not been entirely elucidated [42]. This may explain the differences in the findings of the various studies. However, it has been suggested that high GI diets increase non esterified fatty acid concentrations after intervention compared to baseline, and increased levels of non-esterified fatty acids can cause beta cell dysfunction, insulin resistance, and reduced glucose uptake [31]. In other words, elevated levels of blood glucose, insulin, and free fatty acids following a high GI diet can induce insulin resistance, which could lead to increased triglyceride, a greater inflammatory response, and a decrease in HDL cholesterol [43,44].

Hyperinsulinaemia and insulin resistance are significantly correlated to dyslipidaemia and contribute to the changes in the plasma lipid profile [45]. Therefore, the potential effects of a low GI diet on cardiometabolic parameters may be caused by a reduction of hyperglycaemia, hyperinsulinaemia and levels of free fatty acids, which could lead to a reduced risk of insulin resistance, beta cell dysfunction, dyslipidaemia, and inflammatory response [45,46].

### 4.2. The Effect of a Low GI Diet on Inflammatory Parameters

Based on the findings of the meta-analysis in relation to the inflammatory parameters, low GI diets significantly decreased (*p* < 0.05) levels of interleukin-6 compared with the higher GI diets in patients with type 2 diabetes. Differences between the two groups were not statistically significant (*p* > 0.05) with respect to C-reactive proteins. The mechanism for this finding, with respect to interleukin-6, may due to hyperglycaemia in the higher GI group, which induces the release of inflammatory cytokines from monocytes [47]. Furthermore, the exposure of endothelial cells to varying levels of glucose concentration can increase the risk of oxidative stress and apoptosis and thus lead to the production of pro-inflammatory cytokines [39,48,49]. The results of this review confirm the findings of a previous study by Juanola-Falgaroma et al. [50], which found that subjects allocated a low GI diet showed significantly higher decreases in interleukin–6 after intervention.

This review has both clinical and public health implications in terms of our understanding of the role low GI diets in the management of cardiometabolic and inflammatory parameters in patients with diabetes.

## 5. Limitations

Although a total of nine studies were included in the meta-analysis, this number was limited by the two sub-groups of GDM and type 2 diabetes. More studies in each of the sub-groups would have further enhanced the wider application of the findings of this review. In addition, the lack of a consensus on what constitutes a low GI diet, and the variation in the GI levels of dietary interventions in the studies included, may have impacted the analysis of the findings of this review.

## 6. Conclusions

This systematic review and meta-analysis have demonstrated that there were no significant differences (*p* > 0.05) between low GI and higher GI diets in relation to total cholesterol, HDL, and LDL cholesterol in patients with type 2 diabetes. However, a significant difference (*p* < 0.05) was observed between the two groups with respect to triglycerides in patients with type 2 diabetes. The results of the effect of a low GI diet on the lipid profile in patients with GDM were not consistent. With respect to the inflammatory parameters, the low GI diet significantly decreased interleukin–6 in patients with type 2 diabetes than the higher GI diet. More studies are needed in this area of research.

## Figures and Tables

**Table 1:** Search Strategy and Search Terms.

Population	Intervention	Comparator	Study Designs	Combining Search Terms
Patients with Diabetes	Low Glycemic Index (GI) Diet	Higher GI Diet	Randomised Controlled Trial	
Type 2 diabetes OR diabetes OR Patients with diabetes OR diabetes mellitus OR Gestational diabetes OR gestational diabetes mellitus (GDM) OR gestational diabetes mellitus OR diabetes mellitus, gestational OR diabetes in pregnancy	GI diet OR glycemic index OR Glycemic Index Numbers OR glycemic load OR Glycemic Indices OR Glycemic Index Number		#1 Controlled clinical trial OR Randomised controlled trial OR placebo OR randomized OR groups OR drug therapy OR randomly OR trial	Column 1 AND Column 2 AND Column 3
			#2 “Animals” NOT “Humans”	
			#3 #1 NOT #2	

**Table 2:** Inclusion and Exclusion Criteria Based on PICOS Framework.

	Inclusion Criteria	Exclusion Criteria
Population	Patients with gestational diabetes or patients with type 2 diabetes	Studies involving participants with type 1 diabetes and animal studies. Studies that include children that have diabetes or adults that are healthy. Pre-existing diabetes in patients who are pregnant
Intervention	Low GI diet	Studies involving dietary supplements
Comparator	higher GI diet	Studies involving additional supplements
Outcomes	Primary outcome measures of interest: Cardio-metabolic: total cholesterol, low density lipoprotein (LDL) cholesterol, high density lipoprotein (HDL) cholesterol, triglycerides. Secondary outcome measures of interest: Inflammatory parameters: C–reactive protein, Adiponectin and Interleukin–6	Qualitative outcomes
Types of Study: Quantitative	Randomised controlled trials	Observational studies Letters Comments Reviews Editorials

**Table 3:** The summary of studies selected for the review.

Citation	Country	Type of Diabetes	Length of Study	Study Type	Age (Years)	Sample Size	Interventions/Glycemic Index (GI) Values	Results/Conclusion
Gomes et al. [31]	Brazil	Type 2 diabetes	1 month	Parallel Design	42.4 ± 5.1	*n* = 20	Low GI diet v. higher GI diet (Mean ± SD) Baseline Higher GI: 66 ± 4 Low GI: 63 ± 6 Post-intervention Higher GI: 72 ± 3 Low GI: 54 ± 4	Serum non esterified fatty acid level increased in the higher GI group compared to the low GI group after intervention (*p* = 0.032). Low GI diet prevented the inflammatory responses induced by higher GI diet.
Grant et al. [32]	Canada	GDM	From 28 weeks gestation until delivery	Parallel Design	Higher GI: 34 ± 1.1 Low GI: 34 ± 0.1	Low GI: *n* = 23 Higher GI: *n* = 24	Higher GI v. Low GI (Mean ± SD) Higher GI: 58 ± 0.5 Low GI: 49 ± 0.8	The difference between the low GI and higher GI groups in respect of lipids and CRP were not statistically significant (*p* > 0.05).
Ma et al. [33]	China	GDM	12–14 Weeks	Parallel Design	Higher GI: 30.0 ± 3.5 Low GI: 30.1 ± 3.8 *p* = 0.901	Higher GI: *n* = 42 Low GL: *n* = 41	Higher GI v. Low GI (Mean ± SD) Baseline Higher GI: 56.1 ± 2.4 Low GI: 56.0 ± 2.1 Post-intervention Higher GI: 53.8 ± 2.5 Low GI: 50.1 ± 2.2	The increases in TC, TG and the decrease in HDL cholesterol were significantly lower (*p* < 0.05) in the low GI group compared with the higher GI group.
Jenkins et al. [34]	Canada	Type 2 diabetes	6 months	Parallel Design	(Mean ± SD) High-cereal fibre diet = 61 ± 9 Low-GI diet = 60 ± 10	210	Low GI diet v. high-cereal fibre diet Mean (95% CI) Baseline Higher GI: 81.5 (80.4–82.7) Low GI: 80.8 (79.6–82.0) Post-intervention Higher GI: 83.5 (82.4–84.7) Low GI: 69.6 (67.7–71.4)	HDL cholesterol increased by 1.7 mg/dL in the low GI group and decreased by −0.2 mg/dL in the higher GI group (*p* = 0.005). Reductions of the CRP were similar in the low GI and higher GI groups.
Jenkins et al. [35]	Canada	Type 2 diabetes	3 months	Parallel Design	(Mean±SEM) High-wheat fibre diet: 61 ± 1.0 Low-GI legume diet: 58 ± 1.3	121	Low GI legume diet v. high-wheat fibre diet Mean (95% CI) Baseline Higher GI: 78 (77–80) Low GI: 80 (79–82) Post-intervention Higher GI: 82 (81–83) Low GI: 66 (64–67)	Low GI legume produced significant decreases in TC (*p* < 0.001) and TG (*p* < 0.001) with no significant change in HDL cholesterol (*p* = 0.19). The relative reduction in TC and HDL cholesterol were greater in the low GI legume diet group compared with the higher GI diet group. No other lipid treatment differences were significant.
Ma et al. [36]	USA	Type 2 diabetes	12 months	Parallel Design	(Mean ± SD) 53.53 ± 8.40	40	Low GI diet v. American Diabetes Association diet (ADA) (Mean ± SEM) Baseline ADA: 82.03 ± 1.31 Low GI: 79.35 ± 1.36 Post-intervention ADA: 80.36 ± 1.40 Low GI: 76.64 ± 1.46	There were no significant differences between low GI and higher GI groups with respect to TC, HDL and TG.
Wolever et al. [37]	Canada	Type 2 diabetes	12 months	Parallel Design	(Mean ± SEM) Higher GI diet: 60.4 ± 1.1 Low GI diet: 60.6 ± 1.0	162	Low GI diet v. higher GI diet (Mean ± SEM) Baseline Higher GI: 61.5 ± 0.4 Low GI: 60.3 ± 0.4 Post-intervention Higher GI: 63.2 ± 0.4 Low GI: 55.1 ± 0.4	There were no significant effects for TC. With the low GI diet, mean triacylglycerol was 12% higher, HDL was 4% lower, the higher GI values were intermediate. The CRP with the low GI diet was 29% less than the higher GI diet (*p* < 0.05).
Yusof et al. [38]	Malaysia	Type 2 diabetes	12 weeks	Parallel Design	Not data	104	Low GI diet v. conventional carbohydrate exchange (CCE) (Mean ± SD) Baseline Higher GI: 64 ± 6 Low GI: 63 ± 5 Post-intervention Higher GI: 64 ± 5 Low GI: 57 ± 6	TG increased at week 4, then decreased at week 12 in the Low GI group and this was reversed in the CCE group. Serum HDL cholesterol increased significantly in both groups over time, although no significant differences were found between the two groups.
Argiana et al. [39]	Greece	Type 2 diabetes	12 weeks	Parallel Design	Control: 63.0 ± 1.3 Low GI: 61.3 ± 1.4	*n* = 61	Low GI diet v. Higher GI diet	The differences between the low GI diet and control diet with respect to HDL cholesterol at the end of the study was statistically significant (*p* = 0.007). A significant decrease (*p* = 0.02) in CRP was found in participants in the low GI diet group and the differences between the low GI and the higher GI groups were significant (*p* = 0.007) after the study. Serum IL–6 and adiponectin did not differ significantly in both groups at week 0 and week 12.
Cai et al. [40]	China	Type 2 diabetes	12 months	Parallel Design	56.7 ± 3.5	*n* = 130	Low GI diet v. Higher GI diet	After intervention, the levels of CRP-reactive protein and IL–6 in the low GI diet were significantly lower than the control group (*p* < 0.05).

Abbreviations: CCE (conventional carbohydrate exchange); Higher GI (Higher glycemic index); Low GI (Low glycemic index); *n* (Number); TC: total cholesterol; TG: triglyceride; CRP: C-reactive protein; IL–6: interleukin 6; v. (Versus).

**Table 4:** Cardio–metabolic and inflammatory parameters of studies included.

Citation	Baseline Versus Post-Intervention	HDL Cholesterol	LDL Cholesterol	Total Cholesterol	Triglyceride	C–Reactive Protein	Adiponectin	Interleukin–6
Gomes et al. [31]	Baseline mg/dL Median (Minimum/Maximum) Post-intervention	Higher GI: 43 (30/59) Low GI: 38 (27.6/45.2) Higher GI: 40 (30/54) Low GI: 41 (24.5/47)	No Data	Higher GI: 210.1 (180/273.5) Low GI: 200.4 (123/248.1) Higher GI: 211 (172/284) Low GI: 214.1 (145/288.5)	Higher GI: 180.2 (88.7/287) Low GI: 195 (68/372) Higher GI: 175.3 (132/311.2) Low GI: 205.1 (63/384.1)	(mg/L) Higher GI: 2.6 (0.8/7.3) Low GI: 2.7 (0.5/5.5) Higher GI: 2.8 (0.6/6.13) Low GI: 2.5 (0.1/6.9) *p* = 0.44	ng/mL Higher GI: 30.9 (29.8/31.4 Low GI: 30.1 (29.4/31.3) Higher GI: 30.8 (30.2/31.6) Low GI: 30.5 (26.7/93) *p* = 0.74	No Data
Ma et al. [33]	Baseline mmol/L (Mean ± SD) Post-intervention	Higher GI: 1.96 ± 0.39 Low GI: 1.89 ± 0.33 Higher GI: 1.85 ± 0.36 Low GI: 1.87 ± 0.34	Higher GI: 2.13 ± 0.60 Low GI: 2.19 ± 0.58 Higher GI: 2.16 ± 0.81 Low GI: 2.20 ± 0.54	Higher GI: 5.74 ± 0.74 Low GL: 5.79 ± 1.01 Higher GI: 5.97 ± 0.89 Low GI: 5.96 ± 1.02	Higher GI: 2.20 ± 0.60 Low GI:2.67 ± 1.27 Higher GI: 3.14 ± 1.05 Low GI: 3.09 ± 1.14	No Data	No Data	No Data
Jenkins et al. [34]	Baseline mg/dL (Mean) Post-intervention	Higher GI: 43.1 Low GI: 41.9 Higher GI: 42.8 Low GI: 43.6	Higher GI: 101.1 Low GI: 96.9 Higher GI: 101.3 Low GI: 95.3	Higher GI: 168.4 Low GI: 164.3 Higher GI: 168.4 Low GI: 162.6	Higher GI: 122.0 Low GI: 128.1 Higher GI: 122.2 Low GI: 124.6	Higher GI: 4.59 Low GI: 4.62 Higher GI: 2.82 Low GI: 3.02	No Data	No Data
Jenkins et al. [35]	Baseline mg/dL (95% CI) Post-intervention	Higher GI: 47 (44, 50) Low GI: 43 (40, 46) Higher GI: 48 (45, 52) Low GI: 43 (40, 45)	Higher GI: 91 (81, 101) Low GI: 84 (77, 92) Higher GI: 90 (81, 99) Low GI: 81 (74, 89)	Higher GI: 163 (151, 174) Low GI: 158 (147, 168) Higher GI: 161 (150, 172) Low GI: 149 (139, 160)	Higher GI: 124 (104, 145) Low GI: 149 (125, 173) Higher GI: 115 (96, 133) Low GI: 128 (107, 148)	No Data	No Data	No Data
Ma et al. [36]	Baseline mg/dL (Mean ± SEM) Post-intervention	Higher GI: 42.95 ± 2.26 Low GI: 45.42 ± 2.38 Higher GI: 44.29 ± 2.30 Low GI: 47.53 ± 2.43	Higher GI: 88.95 ± 7.52 Low GI: 93.16 ± 8.07 Higher GI: 71.49 ± 7.81 Low GI: 94.50 ± 8.32	Higher GI: 168.10 ± 9.06 Low GI: 175.58 ± 9.53 Higher GI: 149.71 ± 9.35 Low GI: 173.63 ± 0.06 *p* = 0.09	* Higher GI: 5.05 (0.14) Low GI: 4.99 (0.15) Higher GI: 4.93 (0.15) Low GI: 4.90 (0.16)	No Data	No Data	No Data
Wolever et al. [37]	Baseline mmol/L (Mean ± SEM) Post-intervention	Higher GI: 1.14 ± 0.05 Low GI: 1.21 ± 0.03 Higher GI: 1.19 ± 0.03 Low GI: 1.16 ± 0.03	Higher GI: 2.82 ± 0.13 Low GI: 3.02 ± 0.13 Higher GI: 3.0 ± 0.08 Low GI: 2.92 ± 0.05	Higher GI: 4.86 ± 0.16 Low GI: 5.09 ± 0.13 Higher GI: 5.04 ± 0.08 Low GI: 5.04 ± 0.08	Higher GI: 2.07 ± 0.15 Low GI: 1.87 ± 0.10 Higher GI: 2.0 ± 0.07 Low GI: 2.17 ± 0.07	** Higher GI: 3.34 (2.56, 4.26) Low GI: 2.64 (1.89, 3.70) Higher GI: 2.75 (2.33, 3.24) Low GI: 1.95 (1.68, 2.27)	No Data	No Data
Yusof et al. [38]	Baseline mmol/L (Mean ± SEM) Post-intervention	Higher GI: 1.18 ± 0.34 Low GI: 1.08 ± 0.30 Higher GI: 1.21 ± 0.05 Low GI: 1.14 ± 0.04	Higher GI: 2.78 ± 0.67 Low GI: 2.78 ± 0.67 Higher GI: 2.93 ± 0.14 Low GI: 2.67 ± 0.11	Higher GI: 4.56 ± 0.80 Low GI: 4.54 ± 0.75 Higher GI: 4.80 ± 0.16 Low GI: 4.54 ± 0.12	Higher GI: 1.35 ± 0.53 Low GI: 1.5 ± 0.47 Higher GI: 1.46 ± 0.08 Low GI: 1.59 ± 0.10	No Data	No Data	No Data
Argiana et al. [39]	Baseline mg/dL (Mean ± SEM) Post-intervention	Higher GI: 46.4 ± 1.8 Low GI: 43.1 ± 1.3 Higher GI: 46.1 ± 1.7 Low GI: 43.3 ± 1.2	Higher GI: 104.9 ± 5.1 Low GI: 107.0 ± 5.5 Higher GI: 104.2 ± 5.2 Low GI: 97.2 ± 6.2	Higher GI: 176.6 ± 5.2 Low GI: 173.9 ± 6.4 Higher GI: 175.8 ± 5.2 Low GI: 167.0 ± 4.1	Higher GI: 126.5 ± 10.8 Low GI: 119.2 ± 11.6 Higher GI: 127.5 ± 10.3 Low GI: 122 ± 9.3	*** Higher GI: 2.1 ± 0.5 Low GI: 4.4 ± 1.2 Higher GI: 2.8 ± 0.6 Low GI: 3.0 ± 0.8	*** Higher GI: 7.4 ± 1.6 Low GI: 12.2 ± 3.4 Higher GI: 8.3 ± 2.1 Low GI: 12.5 ± 1.5	**** Higher GI: 1.3 ± 0.2 Low GI: 1.4 ± 0.3 Higher GI: 2.0 ± 0.5 Low GI: 1.3 ± 0.2
Cai et al. [40]	Baseline mg/L (Not stated whether Mean or SD) Post-intervention	No Data	No Data	No Data	No Data	Higher GI: 8.03 ± 0.72 Low GI: 8.04 ± 0.75 Higher GI: 5.01 ± 0.32 Low GI: 3.68 ± 0.29	No Data	**** Higher GI: 12.26 ± 1.57 Low GI: 12.29 ± 1.44 Higher GI: 9.01 ± 0.83 Low GI: 7.97 ± 0.86

Abbreviations: CCE (conventional carbohydrate exchange); Higher GI (Higher glycemic index); Low GI (Low glycemic indexn (Number); TC: total cholesterol; TG: triglyceride; CRP: C-reactive protein; IL–6: interleukin 6. * mmol/L (Natural Logarithm); ** mg/L (Mean, 95% CIs); *** µg/mL (Mean ± SEM); **** pg/mL (Mean ± SEM); v. (Versus).

**Table 5:** Results of a meta-analysis of the effect of a low GI diet on inflammatory parameters.

Outcomes	Patients with Type 2 Diabetes			
	N Studies	Weighted Mean Difference (95% CI) mg/L	*p*-Value	I^2^ %
Interleukin–6	2	−1.01 (−1.55, −0.48)	0.001	0.0
C–eactive Protein	5	−0.32 (−1.17, 0.53)	0.467	0.0
Adiponectin	2	0.01 (−0.00, 0.03)	0.072	0.0
